# A Comparison Between the Effectiveness of Isoamyl 2-Cyanoacrylate Tissue Adhesive and Silk Sutures in Wound Closure Following Minor Oral Surgical Procedures: A Prospective Clinical Study

**DOI:** 10.7759/cureus.41973

**Published:** 2023-07-16

**Authors:** Javed Akhter, Sangita Kalita, Rohit Goyal, Pooja Jaiswal, Karthikeyan Ramalingam, Divya Yadav

**Affiliations:** 1 Oral and Maxillofacial Surgery, Maharaja Ganga Singh Dental College and Research Centre, Sri Ganganagar, IND; 2 Oral Pathology and Microbiology, Saveetha Dental College and Hospitals, Saveetha Institute of Medical and Technical Sciences, Saveetha University, Chennai, IND

**Keywords:** local ulceration, swelling, adhesive, trismus, pain, minor oral surgery, wound dehiscence, silk suture, iso-amyl 2 cyanoacrylate

## Abstract

Background

In order to maintain the surgery site's shape, functionality, and aesthetics, closure of the wound is essential for intra-oral and general surgical procedures. Wound closure speeds up healing by reducing the buildup of inflammatory cells. For a wound to heal well, the incision must be precise, the tissue must be handled delicately, the wound must be precisely repositioned, and the closure material must have optimum functional properties and be sterile.

Aim

This study aims to conduct a clinical comparison of the effectiveness of silk suture versus isoamyl 2-cyanoacrylate (IAC) for intra-oral mucosal incisions.

Methodology

Fifty patients who needed a minor oral surgical operation under local anesthesia from the Department of Oral and Maxillofacial Surgery were recruited for this prospective clinical trial. Ethical clearance and informed consent were obtained for this study. Two groups were created from the sample of 50 patients in this investigation. An intra-oral mucosal incision was closed in one group using a 3-0 silk suture and in the second using two drops of IAC. An accurate approximation of the incised edges was used to avoid leaving any gaps between them. Parameters such as the time taken for closure, pain, bleeding, swelling, mouth opening, wound dehiscence, wound infection, and local ulceration were evaluated in this study. A visual analog scale (0-10) was used to assess the pain score.

Facial swelling was evaluated by the tape method given by Gabka and Matsumura. The measurement was done from tragus to pogonion, tragus to oral commissure, and outer canthus to gonion. The sum of these measurements was calculated. By measuring the distance between the incisal edges of the upper and lower central incisors, the trismus was evaluated using a graduated metal scale. Assessment of bleeding (0-4) was done by asking the patient.

Assessment of wound dehiscence and local ulceration was done based on visual inspection and palpation on the first, second, and seventh days postoperatively. All the recorded parameters were tabulated. The statistical analysis was done using SPSS version 25.0 (IBM Corp., Armonk, NY). The Chi-square test was used to compare the categorical variables between the study groups. The independent t-test was used to compare the means between the study groups. The statistical significance was kept at a p-value less than 0.05.

Results

The results showed that there was no statistically significant difference between suture and IAC in terms of incidence of pain and wound dehiscence. But the time taken for wound closure was less with IAC, and the pain score on the seventh day was higher with IAC and statistically significant.

Conclusion

We observed that IAC was as effective as the gold standard silk suture. The advantages of IAC are its hemostatic and bacteriostatic qualities, and IAC also took less time to complete the procedure.

## Introduction

The use of the proper suturing method and suture material aids in wound closure and aims to preserve the shape, function, and esthetics of the operative site in intra-oral and general surgery procedures [1]. The sutured tissue can resist infection depending on the suture material used [2]. For proper wound healing, precision of the incision should be a must along with proper handling of tissue and re-approximation, and the material used for closure must have optimum functioning properties and be aseptic. Other factors also contribute to good wound healing such as the overall systemic health of the patient, nutritional status and immune responses, and the presence or absence of infection in the wound. Unintentional needle stick injuries increase the surgeon’s risk of developing diseases such as AIDS and hepatitis. The iatrogenic problem of suturing led to the development of tissue adhesive as a way to close the edges of the wounds. Tissue response to the suture material becomes more significant in diabetic and immunocompromised patients [3,4].

Isoamyl 2-cyanoacrylate (IAC), one of the ingredients of tissue adhesives, was developed in 1959. Early tissue adhesives were ethyl and alkyl in composition. Due to their harmful effects on the tissue, these adhesives were discontinued. However, despite having a lengthy molecular chain, IAC is not poisonous and provides benefits such as hemostasis, bacteriostatic characteristics, and adhesive qualities, and it can be used to close wounds and restore organs, mucosa, skin, nerves, and blood vessels [5,6]. From 1996, IAC was authorized for usage in clinical settings. It gained popularity due to its painless and aesthetic closure, lesser application time, and good tensile strength in spite of moist environments [6]. The flexibility of IAC could be increased with the addition of plasticizers, and it is used for the treatment of embolization, varices of the stomach and esophagus, and even intra-cranial arteriovenous malformations [7].

The irritation from the prick of the needle and tearing of the wound margin is entirely eliminated by tissue adhesive polymers, which also close the wound margins. An excellent tissue adhesive should have properties such as good stability, adequate working time, optimum cover area, ease of use, biodegradability, and non-carcinogenicity [4].

The aim of the current study was to compare the clinical effectiveness of silk sutures and IAC on intra-oral incisions.

## Materials and methods

This prospective study was carried out on 50 patients from the Department of Oral and Maxillofacial Surgery at Maharaja Ganga Singh Dental College and Research Centre, Sri Ganganagar, Rajasthan, India. The sample size was calculated using the values obtained in the study by Vastani et al. [3] with G*power software.

An ethical clearance letter was obtained through MGSDC/ECC/19 for this study, and informed consent was obtained from every participant. These patients required minor oral surgical procedures like extraction of teeth under local anesthesia. The inclusion criteria were apparently healthy patients in the age range of 18-35 years whose intra-oral incisions could be approximated without stress. They were selected without regard to gender, religion, and socioeconomic status as well as the history of anesthetic or medication allergies or tobacco usage. The exclusion criteria include patients with systemic illnesses such as blood dyscrasias and immunodeficiency, those receiving anticoagulant treatment, patients with mental disabilities, uncooperative patients, flaps that did not approximate passively, and the presence of any local pathology at the surgical site.

The patients were randomly allotted to both groups by the coin-toss method (heads: silk suture and tails: IAC). It was performed on patients requiring atraumatic extraction of third molars with minimal bone cutting and tissue handling. The Group I patients had wound closure with 3-0 silk suture material (Trusilk SN:5028), and the Group II patients had wound closure with IAC (Amycrylate Bio-Adhesive, Concord Drugs, India).

The surgical site was anesthetized with 2% lignocaine and 1:80,000 adrenaline. The length of the incision varied from 1 to 3 cm depending on the surgical access required for the extraction procedure. After completing the atraumatic surgery and attaining sufficient hemostasis, patients in Group I received 3-0 silk sutures to close the wound, and patients in Group II received IAC after proper isolation.

An accurate approximation of the incised edges was used to prevent gaps between them. For the purpose of closing mucoperiosteal flaps, drops of IAC were administered to the approximate incision margins. Patients received postoperative advice on nutrition and maintenance of wounds including care of the glue site, dental hygiene, and warm saline rinses. The patients received postoperative analgesics (aceclofenac 100 mg with paracetamol 500 mg) as and when needed.

Follow-up was performed on the first, second, and seventh days with visual analog scale (VAS) scoring and facial swelling measurement. VAS score [5] was scored as no pain: 0-1; mild annoying pain: 2-3; nagging uncomfortable pain: 4-5; distressing miserable pain: 6-7; intense, dreadful horrible pain: 8-9; and worst possible, unbearable, excruciating pain: 10. For facial swelling, the tape method was used. The measurement was done from tragus to pogonion (ear to chin), tragus to oral commissure (outer corner of the mouth), and outer canthus to gonion (angle of the mandible). The sum of these measurements was calculated.

The results were recorded in tables and graphically represented utilizing SPSS v25.0 (IBM Corp., Armonk, NY). The statistical analysis was completed using the standard analysis of variance (ANOVA) and t-tests. The statistical significance was kept at a p-value less than 0.05.

## Results

**Figure 1:**
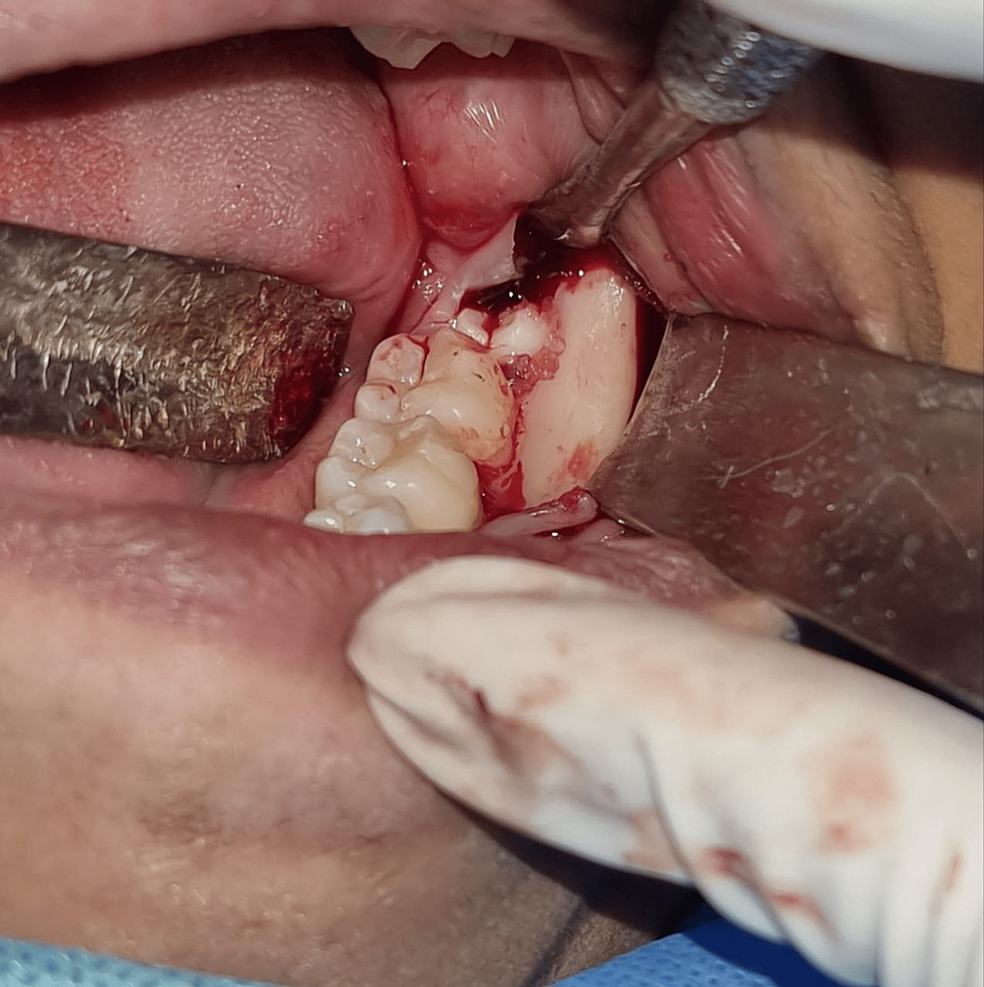
Intraoperative picture Intraoperative picture showing the extraction of the third molar.

**Figure 2:**
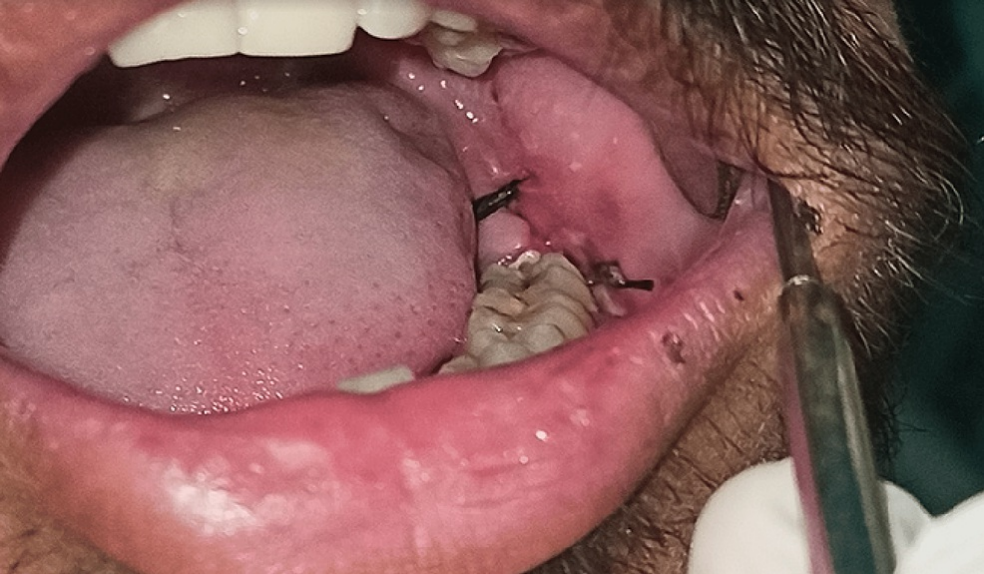
Intraoperative picture showing silk sutures Intraoperative picture of the surgical site closed with 3-0 silk sutures.

This prospective clinical study was done on intra-oral surgical procedures (Figure 1) to evaluate the clinical effectiveness of IAC in the closure of intra-oral incisions when compared with 3-0 silk sutures (Figure 2).

Group I comprised the 3-0 silk suture group. It had 25 individuals comprising 12 males and 13 females in the age group of 19-30 years. Around 72% had bleeding on Day 1, 92% had bleeding on Day 2, and 92% had bleeding on Day 7; 80% had wound dehiscence, 12% showed wound infection, and 96% did not show local ulceration (Table 1).

**Table 1:** Study parameters of Group I (silk suture group) This table shows the parameters and their distribution among Group I (3-0 silk suture).

Variables	Options	Frequency	Percentage
Age	19 to 24 years	14	56.0
	25 to 30 years	11	44.0
	Total	25	100.0
Gender	Male	12	48.0
	Female	13	52.0
	Total	25	100.0
Bleeding - Day 1	Present	18	72.0
	Absent	7	28.0
	Total	25	100.0
Bleeding - Day 2	Present	23	92.0
	Absent	2	8.0
	Total	25	100.0
Bleeding - Day 7	Present	23	92.0
	Absent	2	8.0
	Total	25	100.0
Wound dehiscence	Present	20	80.0
	Absent	5	20.0
	Total	25	100.0
Wound infection	Present	3	12.0
	Absent	22	88.0
	Total	25	100.0
Local ulceration	Present	1	4.0
	Absent	24	96.0
	Total	25	100.0

Group II comprised the IAC application. It had similar age and gender distribution, but 52% had bleeding on Day 1, 76% had bleeding on Day 2, and 88% had bleeding on Day 7; 64% had wound dehiscence, and 32% had wound infection in the IAC group (Table 2).

**Table 2:** Parameters studied in Group II (IAC group) This table depicts the findings and distribution of various parameters studied among Group II (IAC group). IAC: Isoamyl 2-cyanoacrylate.

Variables	Options	Frequency	Percentage
Age	19 to 24 years	14	56.0
	25 to 30 years	11	44.0
	Total	25	100.0
Gender	Male	12	48.0
	Female	13	52.0
	Total	25	100.0
Bleeding - Day 1	Present	13	52.0
	Absent	12	48.0
	Total	25	100.0
Bleeding - Day 2	Present	19	76.0
	Absent	6	24.0
	Total	25	100.0
Bleeding - Day 7	Present	22	88.0
	Absent	3	12.0
	Total	25	100.0
Wound dehiscence	Present	16	64.0
	Absent	9	36.0
	Total	25	100.0
Wound infection	Present	8	32.0
	Absent	17	68.0
	Total	25	100.0
Local ulceration	Present	2	8.0
	Absent	23	92.0
	Total	25	100.0

The average and standard deviation of Group I had preoperative discomfort, mouth opening, and edema of 0.96 ± 1.274, 40.64 ± 8.062 cm, and 310.44 ± 39.140, respectively. The average time needed to close was 3.040 ± 0.9781. The postoperative pain score and swelling were greater with values of 4.68 ± 0.900 and 328.60 ± 41.389. It was significantly reduced on the second and seventh days. The mouth opening was less on the first day (20.36 ± 6.238), and it increased on the second and seventh days (Table 3).

**Table 3:** Descriptive statistics of Group I (silk suture group) This table shows the descriptive findings observed during preoperative, intraoperative, and postoperative stages using 3-0 silk sutures.

Variables		N	Minimum	Maximum	Mean	Std. deviation
Preoperative	Pain	25	0	4	.96	1.274
	Mouth opening	25	25	52	40.64	8.062
	Swelling	25	230	380	310.44	39.140
Intraoperative	Time taken for closure	25	2.0	5.0	3.040	.9781
Postoperative	Pain - Day 1	25	4	6	4.68	.900
	Pain - Day2	25	2	4	2.44	.651
	Pain - Day 7	25	0	2	.08	.400
	Swelling - Day 1	25	263	420	328.60	41.389
	Swelling - Day 2	25	256	395	320.68	38.535
	Swelling - Day 7	25	230	380	311.08	39.915
	Mouth opening - Day1	25	10	32	20.36	6.238
	Mouth opening - Day 2	25	14	40	29.56	6.989
	Mouth opening- Day 7	25	32	52	42.52	6.325

In group 2, preoperative discomfort, mouth opening, and edema had a mean and standard deviation of 1.32 ± 1.520, 40.60 ± 7.643 cm, and 308.48 ± 40.472, respectively. The average time needed to close was 1.092 ± 0.5612, On the first day after surgery, there was more postoperative pain and swelling, which was about 4.68 ± 0.900 and 326.84 ± 41.715. It reduced simultaneously on the second and seventh days. The mouth opening was less on the first day (20.36±6.238), and it increased on the second and seventh days (Table 4).

**Table 4:** Descriptive statistics of Group II (IAC group) This table shows the descriptive findings observed during preoperative, intraoperative, and postoperative stages using IAC for wound closure. IAC: Isoamyl 2-cyanoacrylate.

Variables		N	Minimum	Maximum	Mean	Std. deviation
Preoperative	Pain	25	0	4	1.32	1.520
	Mouth opening	25	25	51	40.60	7.643
	Swelling	25	230	380	308.48	40.472
Intraoperative	Time taken for closure	25	.4	2.0	1.092	.5612
Postoperative	Pain - Day 1	25	4	6	4.68	.900
	Pain - Day2	25	2	4	2.44	.651
	Pain - Day 7	25	0	2	.72	.980
	Swelling - Day 1	25	250	410	326.84	41.715
	Swelling - Day 2	25	255	395	320.36	38.876
	Swelling - Day 7	25	230	380	308.80	41.546
	Mouth opening - Day 1	25	10	32	20.36	6.238
	Mouth opening - Day 2	25	14	40	29.56	6.989
	Mouth opening - Day 7	25	25	52	40.28	7.098

**Figure 3:**
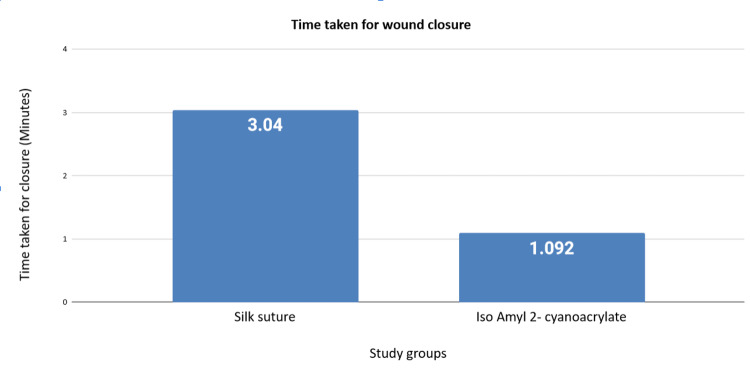
Graphical representation of the mean time taken in minutes between the silk suture group and the IAC group IAC: Isoamyl 2-cyanoacrylate.

Comparison between preoperative pain, mouth opening, and swelling of Group I and II did not achieve statistical significance (Table 5). The time taken for wound closure was a mean of 3.040 minutes for Group I and 1.092 minutes for Group II with a p-value of 0.000, which was statistically significant (Figure 3).

**Table 5:** Comparison between Groups I and II This table shows the comparison of parameters between Group I (silk suture group) and Group II (IAC). The time taken for wound closure and pain on Day 7 was statistically significant (*) among the groups. IAC: Isoamyl 2-cyanoacrylate.

Variables		Study groups	Mean	Std. deviation	p-value
Preoperative	Pain	Silk suture	0.96	1.274	0.405
		Isoamyl 2-cyanoacrylate	1.32	1.520	
	Mouth opening	Silk suture	40.64	8.062	0.986
		Isoamyl 2-cyanoacrylate	40.60	7.643	
	Swelling	Silk suture	310.44	39.140	0.863
		Isoamyl 2-cyanoacrylate	308.48	40.472	
Intraoperative	Time taken for closure	Silk suture	3.040	0.9781	0.000*
		Isoamyl 2-cyanoacrylate	1.092	0.5612	
Postoperative	Pain - Day 1	Silk suture	4.68	0.900	1.000
		Isoamyl 2-cyanoacrylate	4.68	0.900	
	Pain - Day 2	Silk suture	2.44	0.651	1.000
		Isoamyl 2-cyanoacrylate	2.44	0.651	
	Pain - Day 7	Silk suture	0.08	0.400	0.005*
		Isoamyl 2-cyanoacrylate	0.72	0.980	
	Swelling - Day 1	Silk suture	328.60	41.389	0.882
		Isoamyl 2-cyanoacrylate	326.84	41.715	
	Swelling - Day 2	Silk suture	320.68	38.535	0.977
		Isoamyl 2-cyanoacrylate	320.36	38.876	
	Swelling - Day 7	Silk suture	311.08	39.915	0.844
		Isoamyl 2-cyanoacrylate	308.80	41.546	
	Mouth opening - Day 1	Silk suture	20.36	6.238	1.000
		Isoamyl 2-cyanoacrylate	20.36	6.238	
	Mouth opening - Day 2	Silk suture	29.56	6.989	1.000
		Isoamyl 2-cyanoacrylate	29.56	6.989	
	Mouth opening - Day 7	Silk suture	42.52	6.325	0.245
		Isoamyl 2-cyanoacrylate	40.98	7.098	

The comparison of pain on Day 1 and Day 2 was not significant, but pain on Day 7 was a mean value of 0.72 in the IAC group and 0.08 in the silk suture group with a p-value of 0.005 and statistically significant. The swelling measurements on Day 1, Day 2, and Day 7 were not significant.

Comparison of bleeding on Day 1, Day 2, and Day 7, wound dehiscence, and wound infection did not achieve statistical significance (Table 6).

**Table 6:** Comparison between Groups I and II This table compared the bleeding, wound dehiscence, wound infection, and local ulceration between Groups I and II. There was no significant difference between the two groups.

Variables	Options		Group I	Group II	p-value
Bleeding - Day 1	Present	Count	18	13	0.244
		% of Total	36.0%	26.0%	
	Absent	Count	7	12	
		% of Total	14.0%	24.0%	
Bleeding - Day 2	Present	Count	23	19	0.247
		% of Total	46.0%	38.0%	
	Absent	Count	2	6	
		% of Total	4.0%	12.0%	
Bleeding - Day 7	Present	Count	23	22	1.000
		% of Total	46.0%	44.0%	
	Absent	Count	2	3	
		% of Total	4.0%	6.0%	
Wound dehiscence	Present	Count	20	16	0.345
		% of Total	40.0%	32.0%	
	Absent	Count	5	9	
		% of Total	10.0%	18.0%	
Wound infection	Present	Count	3	8	0.171
		% of Total	6.0%	16.0%	
	Absent	Count	22	17	
		% of Total	44.0%	34.0%	
Local ulceration	Present	Count	1	2	1.000
		% of Total	2.0%	4.0%	
	Absent	Count	24	23	
		% of Total	48.0%	46.0%	

## Discussion

**Figure 4:**
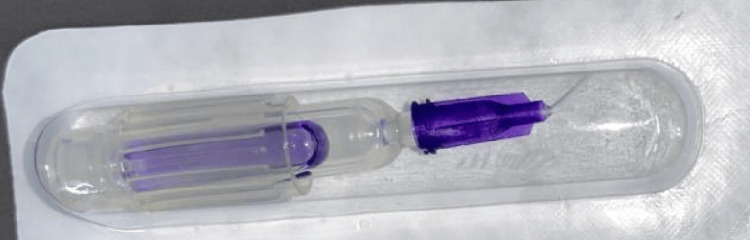
Tissue adhesive The image shows the commercially available tissue adhesive.

Wound healing could be by primary, secondary, or tertiary intention. Suture materials like Vicryl and silk sutures, staples, and tissue adhesives (Figure 4) such as IAC are used to re-adjust the wound edges for primary purposes with faster healing and reduced scarring when opposed to secondary intention [4].

A common practice is a secondary intention that allows the wound to granulate. Normal healing requires more time and results in more scar tissue. During the tertiary intention, the wound is cleaned and debrided for four to five days before it is closed. The key to healing at the surgery site is achieving optimal wound closure [5].

Suture materials show improved tensile strength, decreased dehiscence rate, and appropriate wound closure. There are disadvantages like crosshatched marks, needle penetration of healthy tissue on either side of the wound, tissue reactivity, anxiety, and prolonged treatment. IAC showed faster application time, enhanced bacteriostatic properties, reduced risk of needle stick injuries, increased recovery, and enhanced aesthetics [4-9]. Hence, we performed this study comparing the clinical effectiveness of 3-0 silk sutures and IAC for intra-oral incisions.

In our study, the postoperative pain score was higher on the first day and simultaneously decreased on the second and seventh days for both Groups I and II. The IAC group had higher postoperative pain on the seventh day than Group 1, which was statistically significant. It is in accordance with the literature [10-17]. In our study, Group I experienced higher bleeding on the second and seventh days, but Group II saw reduced postoperative hemorrhage. Howard et al. [18] observed better hemostasis with tissue adhesives [18].

Kulkarni et al. [13] found that using cyanoacrylate resulted in less inflammation and promoted quick initial healing. Kumar et al. [12] compared and contrasted the effects of using black braided silk sutures and IAC to close intra-oral lesions after alveoloplasty. Cyanoacrylate-treated patients experienced rapid hemostasis; however, blood continued to ooze over the sutured site for a brief time after surgery.

In order to examine the use of standard silk sutures and soft tissue glue cyanoacrylate in the mucoperiosteal flap closure following surgical extraction of mandibular impacted third molars, Rewainy et al. [8] reported a significant reduction in hemostasis.

In our research compared to wound infection and wound dehiscence, between Groups I and II, there was no appreciable variation in the prevalence of local ulceration with the exception of two patients in Group 1 who acquired wound infections. In order to compare silk sutures with cyanoacrylate tissue adhesive in the closure of intra-oral wounds, Phani et al. [16] conducted a study where patients treated with cyanoacrylate had a reduced incidence of wound infection than patients whose wounds were closed with 3-0 black braided silk suture. Dalvi et al. [14] also reported a finding similar to our study. Rewainy et al. [8] reported increased wound infection with silk suture material.

In our research, the IAC group required a shorter time for wound closure than the silk suture group, and the result was statistically significant with a p-value of 0.000. Suthar et al. [15] conducted research in which the time length needed for silk suture closure varied from three to seven minutes. In comparison, IAC only took 30 seconds to two minutes to close wounds. When utilizing IAC, hemostasis was accomplished in less than 1.5 minutes as opposed to five minutes when using silk sutures. Dalvi et al. [14] also found that the IAC was swift and saved valuable operating time.

Veríssimo et al. [19] have reported in their systematic review that the use of cyanoacrylate individually or in association with wound dressing agents presents analgesic effects because the patient experienced less pain when cyanoacrylate is applied to the wound closure and covering, thereby reducing the need for postoperative analgesic medication. A literature search for the usage of cyanoacrylates in different clinical applications including pediatric surgeries [20], aphthous ulcers [21], and sinus-lift procedures [22] are reported. Choi et al. [22] have reported the use of adhesives in sinus-lift procedures.

In order to properly immobilize the healing region after a well-planned operation, the wound must be closed using the suitable method and the right materials, such as sutures or tissue adhesives. Silk suture materials can cause tissue injury during the suturing procedure; surgical skill determines tissue tension at the wound site; capillary action by suture material can cause wound infection; and suture removal on the seventh day can be uncomfortable for the patient, resulting in incomplete healing, fistula, and granuloma formation. The perfect tissue adhesive will exhibit shelf stability, complete polymerization even in the presence of moisture (blood, saliva, or water), adequate working time, spread to cover the ideal area, provide wettability, and not generate too much heat during the polymerization process, and provide a strong and flexible bond. It should also be tissue compatible [23].

In our research, both Group 1 and Group 2 experienced a decrease in mouth opening on the first day of roughly 20.36 mm and an increase on the second and seventh days after the procedure. The study is in conflict with that of Rewainy et al. [8] who asserted that there was a difference between the two groups from the first day because patients in the cyanoacrylate glue group had less pronounced trismus. Mahat et al. [23] and Kumar et al. [24] have also reported better efficacy of tissue adhesives [23,24].

Limitations

Our limitation would be the number of individuals per study group, which was 25. A split-mouth study with a larger sample size utilizing the silk suture material and tissue adhesive on the same individual will avoid other variables influencing wound healing. A comparison of different tissue adhesives in different locations in the oral cavity will yield valuable information. A longer follow-up period will also be useful.

## Conclusions

According to our study, suturing and cyanoacrylate adhesive were equally effective in reducing pain in wound closure, but cyanoacrylate was faster in the time taken for wound closure. This study found that intra-oral wound closure with IAC can be done successfully. The process was rapid and comparatively painless, with adequate hemostasis. Larger studies comparing different tissue adhesives will provide valuable information about their clinical efficiency.
